# Pathogenesis of Acute Kidney Injury in Coronavirus Disease 2019

**DOI:** 10.3389/fphys.2021.586589

**Published:** 2021-02-16

**Authors:** Jing-Yi Qian, Bin Wang, Lin-Li Lv, Bi-Cheng Liu

**Affiliations:** Institute of Nephrology, Zhong Da Hospital, Southeast University School of Medicine, Nanjing, China

**Keywords:** coronavirus disease 2019, acute kidney injury, mechanism, SARS-CoV-2, ACE2

## Abstract

Since the outbreak of Coronavirus Disease 2019 (COVID-19) in Wuhan, China, in December of 2019, it has rapidly become a global pandemic. Although acute respiratory disorder is the main manifestation of COVID-19, acute kidney injury (AKI) is another important extrapulmonary complication, which has a critical impact on the prognosis and mortality of patients. Current understanding about the exact pathogenesis of AKI in COVID-19 is unclear. Several studies have suggested that intrarenal, pre-renal and post-renal factors mediated collaboratively by direct virus attack, overloaded immune responses, drugs, sepsis, coagulation dysfunction, and underlying diseases may all be involved in the pathogenesis of AKI. This article reviews the current understanding of the pathogenesis of AKI in COVID-19.

## Introduction

The rapid outbreak of COVID-19 caused by the severe acute respiratory syndrome coronavirus 2 (SARS-CoV-2) that emerged in Wuhan, China, in December of 2019, has rapidly become a potentially life-threatening and economically destructive global pandemic. As of December 26, there have been over 78 million confirmed cases of COVID-19, with 1,740,390 deaths in over 200 countries. COVID-19 mainly manifests as an acute respiratory disease, but it can also affect multiple organs, such as the kidney, heart, blood, nervous system, and gastrointestinal system. Regarding the kidney, this disease can cause hematuria, proteinuria, and even acute kidney injury (AKI) (Chen et al., 2020; Wang D. et al., 2020). The incidence of AKI in COVID-19 varies in different studies, ranging from 0.5 to 36.6% (Guan et al., 2020; Li Z. et al., 2020; Pei et al., 2020). In addition, COVID-19 patients with renal complications show higher mortality (Pei et al., 2020). Autopsy and renal biopsy studies have also confirmed renal injury in some patients with COVID-19, primarily manifested as acute renal tubular necrosis and endothelial injury. Here, we have summarized the pathogenesis of AKI in COVID-19 to help aid the prevention and clinical management of this pandemic.

## Pathogenesis of Acute Kidney Injury in COVID-19

### Direct Attack by the Virus of SARS-CoV-2

Numerous studies have mentioned the incidence of AKI in COVID-19 with different rates of occurrence (Chen et al., 2020; Cheng et al., 2020; Diao et al., 2020; Guan et al., 2020; Hirsch et al., 2020; Huang et al., 2020; Li Z. et al., 2020; Pei et al., 2020; Wang D. et al., 2020), which may be related to the discrepancy of severity and basic conditions of the patients included among studies. AKI is clearly not uncommon in COVID-19. The main clinical manifestations of renal involvement in COVID-19 are proteinuria, hematuria, elevated blood urea nitrogen (BUN) and creatine, and reduced density in computed tomography scan of the kidneys (Cheng et al., 2020; Pei et al., 2020). AKI, defined according to KDIGO criteria, is diagnosed by increases in serum creatinine in and/or falls in urine output in recent literature.

Recent clinical studies and autopsies have provided more evidence that SARS-CoV-2 can directly infect the kidney. According to the published results, AKI in COVID-19 may be induced by viral replication in podocytes and tubules in patients with COVID-19. Diao et al. (2020) analyzed the kidney tissues from six postmortem examinations. Hematoxylin-eosin staining showed severe acute tubular necrosis and lymphocytic infiltration. Immunohistochemistry of tubules showed positive staining for the SARS-CoV-2 nucleocapsid protein antigen. In addition, virus particles were also visible by electron microscopy in two patients. Another study analyzed kidney abnormalities in 26 autopsies of COVID-19 patients, among which nine cases had clinical symptoms of kidney injury. Prominent acute proximal tubular necrosis was observed by light microscopy, with loss of brush border, nonisometric vacuolar degeneration and even frank necrosis. Furthermore, there are apparent virus-like particles in podocytes and the proximal tubular epithelium. However, these were rarely seen in distal tubules or collecting ducts, which only presented with cellular swelling and interstitial dilatation. The difference between proximal tubular and distal tubules may be attributed to the distribution of transmembrane protease serine 2 (TMPRSS2) – the accomplice of SARS-CoV-2. In this study, immunofluorescence staining detected viral nucleoprotein expression in the cytoplasm of the tubular epithelium in three patients (Su et al., 2020). Renal biopsy of a COVID-19 patient with AKI showed collapsing glomerulopathy, and an ultrastructural examination showed a slightly thickened glomerular basement membrane (GBM) with severe foot process effacement, involving over 90% of the GBM. Meanwhile, virus particles existed in podocytes, indicating that SARS-CoV-2 can attack podocytes directly and lead to collapsed glomerulopathy (Larsen et al., 2020). In addition, Peng et al. (2020) detected viral RNA of SARS-CoV-2 from the urine of COVID-19 patients, even in those without urinary symptoms. This confirmed that SARS-CoV-2 can invade the urinary system and enter the urine through glomerular filtration. To conclude, these studies suggested that SARS-CoV-2 can directly infect the kidney to induce AKI.

The targeted attack capability of coronavirus is related to its combined affinity for the targeted cells. There are three main functional receptors for SARS-CoV-2: angiotensin-converting enzyme II (ACE2), CD147 and glucose regulated protein (GRP78). ACE2 has been identified as the cell entry receptor for SARS-CoV-2 (Yan et al., 2020; Zhou P. et al., 2020). The spike glycoprotein of SARS-CoV-2, as the “weapon” of SARS-CoV-2, has been analyzed by cryo-EM to better understanding its trimer structure. Interestingly, spike glycoproteins are “closed” with three downward receptor binding domains, while a single upward domain can initiate the attack of SARS-CoV-2 (Walls et al., 2020; Wrapp et al., 2020). The viral-cell membrane fusion is critical during the attack, which indicates that specific proteolytic cleavage of the spike glycoproteins assists virus infection (Peng et al., 2020). In the kidney, ACE2 is expressed in proximal tubules as well as in podocytes, although the level in podocytes is relatively low (Ye et al., 2006), which provides targets for SARS-CoV-2. Moreover, ACE2 can directly catalyze angiotensin II (Ang II) into angiotensin 1-7 (Ang 1-7), which plays a critical role in anti-fibrotic, anti-proliferative, and anti-inflammatory process in the kidney (Anguiano et al., 2017). And Ang II can also induce kidney injury through various molecular mechanisms. Previous studies have suggested that SARS-CoV-2 can downregulate the expression of ACE2 (Glowacka et al., 2010), affecting the formation of Ang 1-7 to induce renal injury by weakening its protective effect. ACE inhibitors (ACE-Is) or angiotensin receptor blockers (ARBs) were assumed to increase SARS-CoV-2 susceptibility by upregulating ACE-2 expression. However, current researches do not support this hypothesis. There is no correlation between ACE-Is/ARBs and AKI. Meanwhile, neither of them can increase the expression of ACE2 in the human kidney (Jiang et al., 2020; Tetlow et al., 2020). CD147, also named EMMPRIN, is involved in tumor development, plasmodium invasion and virus infection (Castro et al., 2003; Chen et al., 2005; Lu et al., 2018). Wang K. et al. (2020) reported the colocalization of spike protein and CD147 proved by coimmunoprecipitation, ELISA, and immuno-electron microscopy. Meplazumab, an anti-CD147 humanized antibody, could significantly inhibit virus attack and replication. Thus, the CD147-spike protein interaction is an invasive mechanism of SARS-CoV-2. The high expression of CD147 on the basolateral side of the renal tubular epithelium (Kosugi et al., 2015) may partially explains the prominent acute proximal tubular necrosis in patients with COVID-19. Meanwhile, TMPRSS2 in proximal tubules mediates membrane fusion during virus internalization (Walls et al., 2020). Furthermore, a furin-like cleavage site at the spike protein of SARS-CoV-2 facilitates the infection and accelerates this process (Walls et al., 2020). Researchers have revealed that the host cell receptor GRP78 is also an important target for the spike protein of SARS-CoV-2 to assist in the attachment and internalization into host cells. Inhibiting the interaction between the SARS-CoV-2 spike protein and the host cell receptor GRP78 would likely decrease the rate of viral infection (Ibrahim et al., 2020).

Collapsing glomerulopathy has been reported in COVID-19 patients which attracted attention from many researchers. While the cause of collapsing glomerulopathy is not only directly caused by virus infection (Larsen et al., 2020), but also related to genes. Wu et al. (2020) presented six black patients with COVID-19 and apo L1 (APOL1) variant who experienced worsening in renal function and proteinuria rapidly. The renal biopsies of all these patients showed collapsing glomerulopathy without the evidence of direct kidney infection, indicating that APOL1 variant was associated with high-risk of collapsing glomerulopathy in patients with COVID-19 (Wu et al., 2020; Shetty et al., 2021).

Therefore, identifying targets of SARS-CoV-2 and exploring specific inhibitors or antibiotics will help to avoid or alleviate the intrarenal damage mediated by the virus directly. Detecting specific genotype can help clinicians to predict collapsing glomerulopathy and the rates of kidney failure.

### Over Activation of Immune Responses

Severe acute respiratory syndrome coronavirus 2 invasion can activate both innate and adaptive immune responses. Thevarajan et al. (2020) reported the kinetics of the immune response of a patient with mild-to-moderate COVID-19. This research showed increased antibody-secreting cells (ASCs), activated CD4^+^T cells, CD8^+^T cells, immunoglobulin M (IgM) and IgG antibodies and follicular helper T cells (TFH cells) in the blood before symptomatic recovery. These immune cells and antibodies persisted for at least 7 days even after full resolution of symptoms, indicating that a strong early adaptive immune response is crucial to the prognosis of patients. However, many severe and critical patients with COVID-19 experienced lymphopenia, and their adaptive immune response cannot be effectively activated. Most of these patients showed elevated pro-inflammatory cytokines and chemokines, including interleukin-6 (IL-6), interleukin-2 (IL-2), interleukin-8 (IL-8), interleukin-17 (IL-17), granulocyte colony-stimulating factor (G-CSF), granulocyte-macrophage colony-stimulating factor (GM-CSF), monocyte chemotactic protein-1 (MCP1), macrophage inflammatory protein 1α (MIP1α), tumor necrosis factor (TNF), etc. (Cao, 2020; Wang D. et al., 2020).

Coronavirus infection results in the activation of monocytes, macrophages, and dendritic cells to release cytokines, especially IL-6, which instigates an amplification cascade in lymphocytes and endothelial cells by *trans* signaling like *cis* signaling to form cytokine storm (Moore and June, 2020). In the kidney injury model mediated by nephrotoxicity, IL-6 correlates with the onset and severity of AKI and aggregate renal injury by recruiting neutrophils (Nechemia-Arbely et al., 2008). It is also capable of damaging tight junction structure to affect the endothelial barrier (Desai et al., 2002). The increased endothelial permeability mediated by IL-6 may result in microcirculatory disturbances to induce AKI. It has been shown that TNF-α can bind with tubular receptors to induce apoptosis during AKI (Ramesh and Reeves, 2003). Along with cytokines, immune cells are also involved in the pathogenesis of AKI. Researchers have proved that T cells can induce kidney injury directly in mouse models, while T lymphocyte deficiency can alleviate kidney injury (Liu et al., 2006). Monocytes will increase significantly and differentiate into different subpopulations after renal injury, among which activated M1 macrophages can release proinflammatory factors, chemokines, and nitric oxide synthase to aggravate kidney injury (Huen and Cantley, 2015). In addition, formation of the terminal complement components formation induced by SARS-CoV-2 can recruit neutrophils, monocytes and macrocytes to accelerate vascular inflammation. Besides, it can also stimulate the release of IL-8, MCP-1, etc. to aggravate vascular injury and activate platelets directly to cause platelet aggregation and the release of procoagulant microparticles (PMP). All of these vascular injuries induced by activated complements lead to vascular dysfunction, which may be the inducer of AKI (Noris et al., 2020). The membrane attack complex (MAC), produced by complement activation, can damage both vascular and glomerular endothelial cells (Noris et al., 2020). These tubule injuries caused by SARS-CoV-2 will be directly or indirectly sensed by the innate immune receptors on tubule epithelial cells to form an amplification loop to aggravate kidney injury (Liu et al., 2018).

The autopsy study with COVID-19 patients showed lymphocyte infiltration (Diao et al., 2020; Su et al., 2020) and endothelial damage in the kidney (Su et al., 2020). Immunohistochemical staining showed the infiltration of CD68^+^ macrophages in tubulointerstitium, and two patients showed the infiltration of CD4^+^T cells, CD8^+^T cells, and CD56^+^ natural killer cells, indicating that tubular injury may be induced by the recruitment and infiltration of immune cells. More noteworthy, the complement membrane attack complex (C5b-9) existed in renal tubules from six cases in this report, providing more evidence for the role of the immune response in COVID-19 with AKI (Diao et al., 2020). Therefore, the immune response is associated with AKI in COVID-19.

An overloaded immune response can induce inflammation that leads to acute interstitial nephritis and can also cause acute tubular necrosis induced by cytokine storm. Last but not least, overloaded immune response is also responsible for the endothelial injury that may result in a thrombus. These mechanisms suggest that immune interventions, such as antivirals and antibodies from convalescent plasma of COVID-19 patients can be used for treatment. In addition, adoptive T cell therapy can be used to restore effective antiviral immunity against COVID-19 considering the involved mechanisms (Leen et al., 2006; Caccamo et al., 2020). Relative clinical trials (NCT04351659, NCT04401410) are ongoing, however, clinicians should be aware of the side effects, including cytokine storm. Vaccines targeting the spike protein of SARS-CoV-2 such as adenovirus type-5 (Ad5-nCoV) have been testified the safety and tolerability after administration a single dose in healthy individuals (NCT04313127).

### Abnormal Coagulation

Researchers have found that many patients who died of COVID-19 experienced disseminated intravascular coagulation (DIC), with increased concentrations of D-dimer and other fibrin degradation products, which are correlated with worse prognosis (Zhou F. et al., 2020). Splenic infarction (Xu et al., 2020) and hematuria (Cheng et al., 2020) have also been observed, indicating the occurrence of microangiopathy. Clinicians have also noticed that there is a higher incidence of circuit clotting in dialysis patients with SARS-CoV-2 infection.

Hypercoagulability can be induced by activated coagulation factors, tissue factors, pathogen- associated molecular patterns and damage-associated molecular proteins released during virus infection (Delvaeye and Conway, 2009). Oxidative stress and inflammation can be enhanced by aggregated erythrocytes to induce microvascular injury (Frimat et al., 2013). Whether SARS-CoV-2 can infect vascular endothelial cells directly to cause damage still needs further investigation. The thrombosis in microvascular and DIC are notable in autopsies of COVID-19 patients (Tang et al., 2020). Occasional hemosiderin granules and pigmented casts were identified in the tubular epithelium in kidneys of four COVID-19 patients with hematuria. There were also prominent erythrocyte aggregates obstructing the lumen of capillaries, indicating that abnormal coagulation may participate in the pathogenesis of AKI in COVID-19 (Su et al., 2020).

The hypercoagulable state of COVID-19 patients has been observed due to the accumulating incidence of venous thromboembolism, pulmonary embolism, etc. (Tang et al., 2020). There is convincing evidence from numerous studies that hospitalized patients should receive anti-coagulation treatment recommended by guideline (Barnes et al., 2020), certainly anticoagulant therapy is necessary in COVID-19 patients, especially critical ones. However, the specific dose of anti-coagulation medication remains unclear, and further randomized trials (NCT04344756, NCT04359277) are underway.

### Sepsis

According to a report from clinical studies, many severe or critically patients with COVID-19 experienced clinical shock with or without hypotension, manifested as cold extremities, weak peripheral pulses, etc. Many of them developed into metabolic acidosis, dysregulated immune response, coagulation dysfunction, and multiple organ dysfunctions. These patients could be diagnosed with sepsis and septic shock (Singer et al., 2016). Accordingly, some experts proposed that viral sepsis is critical in the pathogenesis of COVID-19. In severe and critical ill COVID-19 cases, the integrity of the epithelial-endothelial barrier is interrupted. To fight against SARS-CoV-2 infection, immune cells and epithelial cells produce multiple proinflammatory cytokines and chemokines to recruit more immune cells to the infection site, leading to uncontrolled inflammation and subsequent viral sepsis (Li H. et al., 2020).

Septic AKI is a common complication of severe and critically ill patients (Vincent et al., 2006), and it is induced by changes in renal hemodynamics, activation of immune cells, massive release of inflammatory molecules, and endocrine dysregulation (Dellepiane et al., 2016). Early research based on endotoxin has suggested that hyperdynamic sepsis is associated with remarkable renal vasodilatation and reduced renal blood flow (Langenberg et al., 2006). However, recent studies have found that AKI and increased renal blood flow occur simultaneously (Langenberg et al., 2005), which may be related to the loss of glomerular filtration pressure due to larger dilatation of renal efferent arterioles than afferent arterioles (Prowle et al., 2012). The redistribution of blood flow, ischemia and medullary hypoxia in sepsis are also causative factors of kidney injury (Bellomo et al., 2017). In addition, cytokines, chemokines, and complements can mediate injury and induce apoptosis in tubular cells during tubular ultrafiltration (Dellepiane et al., 2016). Therefore, various elements lead to viral sepsis and end up with AKI, including direct virus attack on the kidney, abnormal immune response, and microcirculation dysfunctions.

The precise time to initiate renal replacement treatment and the ideal form of blood purification therapy remain controversial. To be sure, it is important to avoid potentially nephrotoxic agents and apply careful fluid and hemodynamic management in patients with septic AKI (Kellum et al., 2020).

### Rhabdomyolysis

Trauma, drugs, toxins, and certain infections are inducers of acute rhabdomyolysis, and AKI is a dangerous complication of severe rhabdomyolysis due to the myoglobin released by damaged cells (Bosch et al., 2009). A severe patient with COVID-19 was reported to be complicated with rhabdomyolysis with lower limb pain and fatigue. The urine analysis revealed that occult blood was positive and urine protein was suspiciously positive. Besides, elevated myoglobin, creatine kinase, lactate dehydrogenase, alanine aminotransferase, and aspartate aminotransferase were observed in his laboratory examination (Jin and Tong, 2020). Upon autopsy, both hemosiderin deposition and pigment casts were observed in tubules, which further confirmed rhabdomyolysis happens in AKI caused by COVID-19 infection (Su et al., 2020).

Thus, rapid clinical diagnosis of rhabdomyolysis is of great significance to avoid the occurrence of AKI or worse prognosis. It is critical to monitor the level of creatine kinase and symptoms such as myalgias and weakness, which assist in the early detection of rhabdomyolysis. Volume expansion, monitoring urine output and correction of mannitol and bicarbonate are necessary for treatment.

### Drugs

The application of norepinephrine, monoclonal antibodies, multiple antiviral drugs, and antibiotics can induce AKI in COVID-19 patients due to the toxicity of drugs during metabolism and excretion by the kidneys (Perazella, 2019). Many drugs have been reported to have renal adverse events on patients (Izzedine et al., 2020), as summarized in Table 1.

**TABLE 1:** Potential AKI causing drugs applied in COVID-19, injury manifestations and related mechanism.

Drugs		Injury	Mechanism
Antivirals	Remdesivir	AKI	Potential mitochondrial toxicity
	Lopinavir/ritonavir	ATN	Transporter defects Apoptosis; Mitochondrial damage
	Interferon	ATN FSGS	Direct proximal tubule cytotoxicity Podocyte injury Enhanced cellular immunity
	Acyclovir	Nephrolithiasis	Intratubular crystal deposition Direct tubular cytotoxicity
Antibiotics	Aminoglycosides	ATN	Direct tubule cytotoxicity
	Amphotericin B	Renal tubular acidosis Necrosis of tubular epithelial cells	Transporter defects Cytotoxicity
	Fluoroquinolones	AIN Tubular damage	Vasoconstriction and thrombotic microangiopathy
	Vancomycin	ATN AIN	Direct proximal tubule cytotoxicity
	β-lactam	ATN Acute glomerulonephritis	Transporter defects Direct proximal tubule cytotoxicity
Aminoquinoline family	Chloroquine	Podocytopathy Mimicking Fabry Disease	Accumulation in lysosomes and induced phospholipidosis
	Hydroxychloroquine		
Monoclonal antibodies	Adalimumab	Nephritis and renal failure	Cytokines Increased capillary permeability Decreased effective circulating blood volume
Others	Furosemide	AKI	Pre-renal azotemia Aciduria Immune-mediated interstitial inflammation
	Adrenaline Norepinephrine Dopamine	AKI	Pre-renal azotemia
	Cyclosporine	ATN, CKD, proteinuria	Vasoconstriction Thromboses Tubular toxicity Interstitial fibrosis
	NSAIDS	AIN Glomerular injury	Affect renal blood flow Injury caused by metabolites Immune-mediated interstitial inflammation

*AKI, acute kidney injury; ATN, acute tubular necrosis; FSGS, focal segmental glomerular sclerosis; AIN, acute interstitial nephritis; CKD, chronic kidney disease.*

Drug nephrotoxicity can destroy renal tubules and cause interstitial damage. Thus, more attention must be paid to drug selection and adjusting the dosage when necessary, especially for those with chronic kidney disease.

### Underlying Diseases

A prospective cohort study evaluated the association between kidney disease on admission and in-hospital mortality of COVID-19 patients. Patients with elevated baseline serum creatinine (11.9%) showed a higher incidence of AKI than those with normal baseline values (4.0%). Moreover, patients with prevalence of kidney disease also had significantly higher in-hospital mortality (Cheng et al., 2020). Thus, renal function indicators need to be closely monitored especially for those with renal abnormalities. In addition, diabetes mellitus, cardiovascular disease, and hypertension are also risk factors of AKI in COVID-19 (Hirsch et al., 2020).

## Potential Therapies

Recently the 25th Acute Disease Quality Initiative (ADQI) Workgroup published the consensus report about COVID-19-associated acute kidney injury (Nadim et al., 2020). Various standard measures including hemodynamic optimization, fluid management, nephrotoxin management, et al. can prevent and manage the risk and stage of AKI. Although antivirals, immunomodulatory agents and systemic anticoagulation help to inhibit the development of AKI according to respective mechanisms, limited data can support the therapeutic effect of them. Anti-inflammatory drugs like statins and nonsteroidal anti-inflammatory drugs (NSAIDs) are considered to have certain anti-inflammatory and potential immunomodulatory effects, but its role in COVID-19 is still unknown. The therapeutic effects of serine inhibitors and recombinant ACE2 that targets the “principal offender and accomplice” of SARS-CoV-2 are still under investigation.

## Conclusion

**FIGURE 1:**
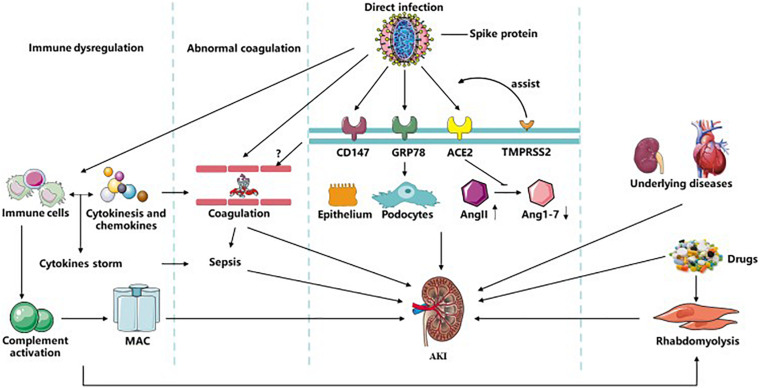
Related mechanisms of AKI in COVID-19. SARS-CoV-2 could infect kidney tissues by ACE2, CD147, and GRP78 with the assistance of TMPRESS2 and furin-like cleavage on spike protein directly to induce kidney injury. It can also affect immune system and complement system, during which MAC, various immune cells, cytokine storm and sepsis were formed and activated resulting in AKI. Abnormal coagulation could be induced by immune system or possible direct injury to vascular caused by virus and ended up with AKI. And rhabdomyolysis induced by infection or drugs participate in the occurrence of AKI. Besides, multiple drugs applied in COVID-19 and underlying diseases of patients like CKD are also causes of COVID-19 with AKI. AKI, acute kidney disease; MAC, membrane attack complex; TMPRSS2, transmembrane protease serine 2; ACE2, angiotensin converting enzyme II; CKD, chronic kidney disease.

COVID-19 is highly infectious and has rapidly developed into a global pandemic rapidly. AKI, as a serious complication of COVID-19, has a strong impact on the prognosis of patients. Several mechanisms are involved in the pathogenesis of AKI in COVID-19, including direct virus invasion, immune dysfunction, abnormal coagulation, sepsis, drugs, and underlying diseases (summarized in Figure 1). These factors mediate AKI directly or indirectly by various molecular mechanisms, which have inspired us several key points of clinical management. First, hemodynamic stability and avoidance of nephrotic agents can decrease the incidence of AKI. Second, regular detection of serum creatine, BUN and creatine kinase can contribute to the early detection of AKI. Lastly, various treatments targeting pathogenesis mechanisms such as receptor antibodies, adoptive T-cell therapy, and anticoagulation therapy, require more attention and research to combat SARS-CoV-2.

## Author Contributions

B-CL designed this study. J-YQ carried out this study. J-YQ and BW wrote the draft. B-CL, L-LL, and BW revised the manuscript. All authors contributed to the article and approved the submitted version.

## Conflict of Interest

The authors declare that the research was conducted in the absence of any commercial or financial relationships that could be construed as a potential conflict of interest.
